# Assay for galactose-deficient IgA1 enables mechanistic studies with primary cells from IgA nephropathy patients

**DOI:** 10.2144/btn-2018-0042

**Published:** 2018-08

**Authors:** Colin Reily, Dana V Rizk, Bruce A Julian, Jan Novak

**Affiliations:** 1 University of Alabama at Birmingham, Department of Medicine, Birmingham, AL, USA;; 2 University of Alabama at Birmingham, Department of Microbiology, Birmingham, AL, USA

**Keywords:** autoimmunity, chemiluminescence, colorimetric assay, ELISA, galactose-deficient IgA1, glycosylation, IgA nephropathy, lectin

## Abstract

**Aims:**

IgA nephropathy, the most common primary glomerulonephritis worldwide, is characterized by glomerular deposition of galactose-deficient IgA1 and elevated serum levels of this IgA1 glycoform. Current ELISA methods lack sensitivity to assess galactose deficiency using small amounts of IgA1, which limits studies in primary cells due to modest IgA1 production in isolated peripheral-blood lymphocytes.

**Methods:**

Lectin from *Helix pomatia* was conjugated to biotin or acridinium ester and used in ELISA to detect galactose deficiency of IgA1 using small amounts of IgA1.

**Results:**

Lectin conjugated to acridinium had an approximately log-fold increased sensitivity compared with biotin-labeled lectin.

**Conclusions:**

The new method of using lectin from *Helix pomatia* conjugated to acridinium increased assay sensitivity, allowing future mechanistic studies with cultured primary cells.

## METHOD SUMMARY

Galactose-deficient IgA1, the key autoantigen in IgA nephropathy, has been difficult to quantify at low IgA1 concentrations. This challenge represents a limitation in researching mechanisms of production of the autoantigen by primary cells, due to the very low number of IgA1-producing cells in blood and, thus, concentration of IgA1 protein in cell culture media. We developed a new chemiluminescence-based quantitative assay with increased sensitivity for detection of galactose-deficient IgA1, even at low IgA1 concentrations produced by cultured mononuclear cells isolated from peripheral blood.

IgA nephropathy (IgAN) is the most common primary glomerulonephritis in the world and a common cause of end-stage renal disease [1]. IgAN is characterized by glomerular immuno-deposits with IgA1 [2]. These immuno-deposits are enriched for IgA1 with galactose-deficient hinge-region *O*-linked glycans (Gd-IgA1) and usually contain complement C3 with variable co-deposits of IgG and/or IgM [2–5]. IgG isolated from these immunodeposits binds Gd-IgA1, confirming the postulate that Gd-IgA1–IgG immune complexes represent a driving force in IgAN pathogenesis [6–14]. Elevated circulating levels of Gd-IgA1 and IgG autoantibodies specific for Gd-IgA1 in IgAN patients each predict disease progression [8,15]. These findings led to the proposal of a multi-hit hypothesis wherein elevated levels of Gd-IgA1 in circulation of IgAN patients induce synthesis of IgG-autoantibodies specific for Gd-IgA1, resulting in the formation of circulating immune complexes, some of which deposit in glomeruli, inciting renal injury [1,9,11].

Human IgA has two subclasses, IgA1 and IgA2. IgA1 has a unique hinge region connecting the Fd and Fc regions of the heavy chains. This hinge region, rich in Ser and Thr residues, has multiple *O*-glycans (3–6 *O*-glycans per hinge region) [16–18]. These glycans are of core 1 type (i.e., *N*-acetylgalactosamine [GalNAc] with β1,3-linked galactose). Core 1 glycans can be sialylated on one or both sugars. Compared with healthy controls, patients with IgAN have more galactose-deficient IgA1 in circulation, that is, IgA1 with some *O*-glycans consisting of terminal GalNAc or sialylated GalNAc. Terminal GalNAc is required for IgG autoantibody binding to Gd-IgA1, but more work is necessary to dissect the role of Gd-IgA1 microheterogeneity in the pathogenesis of the disease [6,7,16].

Mechanisms responsible for elevated Gd-IgA1 production in IgAN remain elusive, but cellular, biochemical and genetic studies have revealed connections with dysregulated expression and activities of specific glycosyltransferases and identified risk-associated loci related to innate and adaptive immune responses [19]. Serum levels of Gd-IgA1 are heritable [20]. EBV-immortalized IgA1-producing cells provided a new tool for analysis of glycosylation pathways related to production of Gd-IgA1 [21]. These studies revealed dysregulation of key enzymes involved in IgA1 *O*-glycosylation, such as glycoprotein-*N*-acetylgalactosamine 3-β-galactosyltransferase 1 (C1GalT1) and its chaperone C1GalT1C1 (Cosmc) that are needed for the addition of galactose to GalNAc in the synthesis of the *O*-linked glycans in the IgA1 hinge region. These conclusions were confirmed by siRNA knockdown of *C1GALT1* and *COSMC* genes [22].

Immortalized IgA1-producing cell lines have been used to assess the effect of cytokines on Gd-IgA1 production. These studies revealed that IL-6 increased Gd-IgA1 production in cells from IgAN patients but not healthy controls [23]. This observation is of great interest, as IgAN patients often have elevated levels of circulating IL-6 and some investigators have suggested a connection to disease progression [24–26]. Studies with immortalized IgA1-producing cells revealed that some cytokines modulate expression of specific glycosyltransferase genes and thereby enhance production of Gd-IgA1[23,27,28]. This effect is due, in part, to an increased and prolonged signaling response to specific cytokines, such as IL-6[28].

These findings from studies using immortalized cell lines need to be confirmed and extended in experiments with primary IgA1-producing cells. We currently lack a sensitive Gd-IgA1 assay for samples with small amounts of IgA1. Peripheral blood has few IgA1-secreting cells and, thus, cell cultures of peripheral-blood mononuclear cells (PBMCs) produce modest quantities of IgA1. To address this problem, we developed a new chemiluminescence assay for Gd-IgA1, using a GalNAc-specific lectin from *Helix pomatia* (HPA) conjugated to an acridinium ester. Different lectins have been used for detection of Gd-IgA1 based on their specificity for terminal GalNAc, including *Helix aspersa* agglutinin (HAA). We have found that the currently available HAA binds less Gd-IgA1 than does the HAA we have purchased previously. We switched to an in-house biotin-labeled HPA that has provided consistent Gd-IgA1 reactivity in ELISA. Here, we demonstrate that the latter lectin, labeled with acridinium, has an increased sensitivity that enables studies with primary cells and small amounts of IgA1.

## Materials & methods

### HPA conjugation with biotin or acridinium

HPA from Sigma Aldrich (L3382–1MG, MA, USA) was conjugated either with biotin (Thermo Fisher Scientific, EZ-Link Sulfo-NHS-LC-biotin, #21327, MA, USA) or acridinium (Cayman Chemical, acridinium NHS ester, #200200, MI, USA). Biotin conjugation was performed as follows: on ice, 1mg HPA was dissolved in 1ml of sterile PBS (pH=7.4) in a glass vial. Next, 143μl of 1mg of NHS-biotin reconstituted in 180μl of H_2_O was immediately added to 1ml of the HPA solution (100mol biotin/mol HPA) and incubated for 30min at room temperature with gentle agitation. After the reaction, buffer was exchanged four times with sterile PBS using a 3-kDa cut-off 15-ml centrifugal concentrator (Amicon Ultra-4, #UFC800324, Millipore) to a final volume of 1 ml for 1 mg of HPA-biotin. Acridinium conjugation was performed as follows: on ice, 1mg of HPA was dissolved in a mixture of 150μl sterile PBS (pH=7.4) and 50μl of 1M sodium bicarbonate (pH=8.75). Solution of 5mg/ml of acridinium in DMSO was prepared and 2μl of acridinium solution was added to HPA solution (100 mol acridinium/mol HPA) and incubated for 20min at room temperature with gentle agitation. Buffer was exchanged four times with sterile PBS using a 3-kDa cut-off 15-ml centrifugal concentrator to a final volume of 1ml for 1mg HPA–acridinium.

### ELISA plates for HPA & IgA assays

Pierce white opaque 96-well plates from Thermo Fisher Scientific (#15042) were used for ELISA with chemiluminescence detection and clear flat-bottom immune 96-well plates for colorimetric ELISA (Thermo Fisher Scientific, #439454). Plates were coated overnight at room temperature with 100μl/well solution of 2.5μg/ml (1.0μg/ml for IgA assay) F(ab′)_2_ fragment of goat IgG specific for α-chain of human IgA (Jackson Immuno Research, #109–006–011, PA, USA) in sterile PBS with 0.05% azide. The following day, plates were washed with PBS and blocked using 1% BSA in PBST (PBS with 0.01% Tween-20) at 200μl/well for 2h at room temperature and then stored at −20°C.

### ELISA protocol for Gd-IgA1 assay

**Figure 1:**
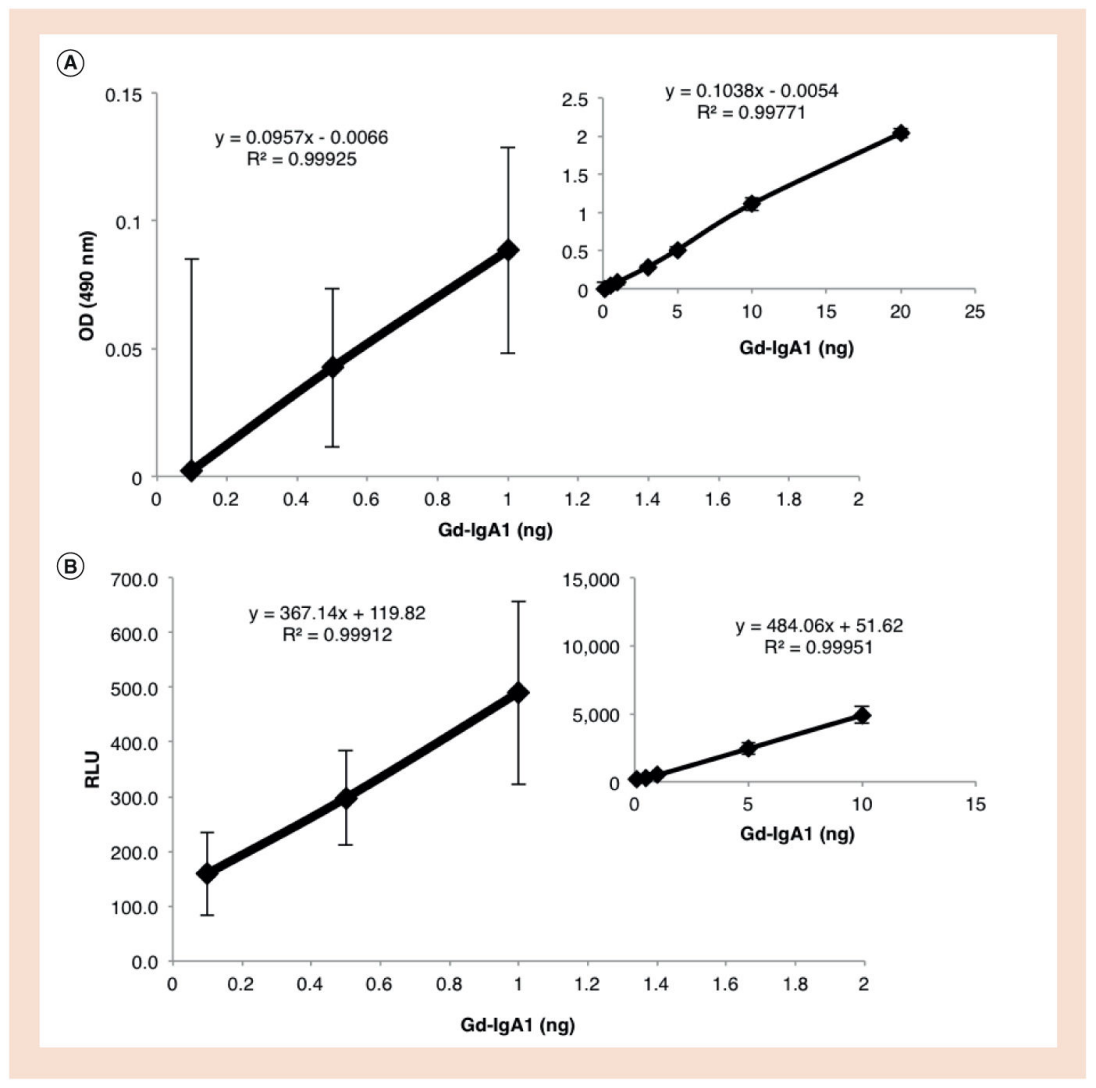
Dynamic ranges of colorimetric and chemiluminescence assays for Gd-IgA1 using lectin from *Helix pomatia* . **. (A)** Standard Gd-IgA1 was incubated at a dynamic range of 0.1–20 ng/well and detected using colorimetric assay. Data were corrected for background signal. The inset shows the entire range with an R^2^ of 0.998. The larger panel is magnified to show the three lowest amounts of Gd-IgA1 (0.1, 0.5 and 1.0 ng). **(B)** Standard Gd-IgA1 was incubated at a dynamic range of 0.1–10 ng/well, and developed using chemiluminescence assay. Data were corrected for background signal. The inset shows the entire range with an R^2^ of 0.999. A portion of the insert has been magnified to show the three lowest amounts of Gd-IgA1 (0.1, 0.5 and 1.0 ng). Error bar goes below 0 in panel A at 0.1 ng. N = 16. Gd-IgA1: Galactose-deficient IgA1; OD: Optical density; RLU: Relative light units.

Serial dilutions of samples and Gd-IgA1 standard were loaded on the plates, diluted in 1% BSA in PBST buffer (100μl/well) and incubated overnight at 4°C. Galactosedeficient IgA1 protein used in this assay was isolated from plasma of a patient with IgA1 myeloma, as previously described [29]. Serial dilutions of standard Gd-IgA1 in 1% BSA in PBST buffer were used to generate a standard curve (Figure 1A & B). Plates were washed with PBS and the captured IgA1 was incubated with sialidase A (100 μl/well) in a humidified chamber at 37°C for 2 h (Prozyme, #GK80040, CA, USA). Plates were washed with PBS and then incubated with HPA–biotin (1:100 dilution) or HPA–acridinium (varied dilutions) in a humidified chamber at 37°C for 3 h (100 μl/well). For HPA–acridinium, plates were washed first with PBST and then with PBS, before reading on a Biotek Synergy H1 using acridinium trigger solutions (Enzo, #ADI-906–001, NY, USA). Chemiluminescence intensity was assessed using 75 μl/well for trigger solution 1 and 2, with 1-s integration; data were reported as relative light units (RLU). For HPA–biotin, plates were washed with PBST, then a 1:2000 dilution of ExtrAvidin–peroxidase conjugate (Sigma #E2886) in 1% BSA in PBST buffer was added (100 μl/well) and incubated in a humidified chamber at 37°C for 1 h (Sigma, ExtrAvidin–peroxidase, #E2886). Plates were washed with PBS and developed using o-phenylenediamine dihydrochloride and H_2_O_2_ for 30 min (100 μl/well), followed by stop buffer (5% sulfuric acid, 100 μl/well). Colorimetric-detection plates were read using Biotek EL808 Ultra microplate reader; data reported as optical density (OD) at 490 nm. Reported units of Gd-IgA1 (U) were based on the regression analysis of Gd-IgA1 standard curve.

### ELISA protocol for IgA assay

Samples and IgA standard were loaded on the plates, serially diluted 1:1 in 1% BSA in PBST buffer (100 μl/well), and incubated overnight at 4°C. Serial dilutions of standard IgA (Luiquichek #592, CA, USA) were used to generate a calibration curve. Plates were washed with PBS and a 1:40,000 dilution of goat anti-human IgA F(ab′)_2_ IgG biotinconjugated antibody (Genway #25–787–278159, CA, USA) in 1% BSA in PBST buffer was added (100 μl/well) and incubated in a humidified chamber at 37°C for 3 h. Plates were then washed, incubated with ExtrA-vidin–peroxidase, developed and read as outlined above in the colorimetric Gd-IgA1 assay.

### Cell cultures

Human subjects provided written informed consent for use of their biological material as approved by the University of Alabama at Birmingham (USA) Institutional Review Board (#150511007). EBV-immortalized IgA1-producing cells from two IgAN patients and four healthy controls and PBMCs from five healthy-control donors were cultured in 1640 RPMI medium with 10% FBS with penicillin (50 U/ml)/streptomycin (50 μg/ml) (Gibco #1507–063) in a humidified incubator with 5% CO_2_ at 37°C. PBMCs were isolated from 15 ml of peripheral blood by centrifugation at 2000 × g for 5 min in heparin vacutainers. Buffy coats were removed and mixed with PBS (up to 10 ml) in a 15-ml conical tube, followed by layering on top of lymphocyte-separation media (4 ml). The tubes were centrifuged at 500 × g for 30 min, followed by cell removal and multiple centrifugation washing steps with PBS. Cell number and viability were assessed using acridine orange/propidium iodide (Logos, #F23001, VA, USA) on a Luna FL (Logos). EBV-immortalized cells were seeded at a concentration of 0.4 × 10^6^ cells/ml in six-well plates at a final volume of 1.5 ml and cultured for 72 h. PBMCs were cultured at concentrations ranging from 11.2 × 10^6^ to 22.2 × 10^6^ cells/ml in a 24-well plate at a final volume of 0.5 ml for 72 h. After incubation, cells were spun at 210 × g for 6 min at 4°C. Medium supernatants were removed and analyzed for IgA1 and Gd-IgA1.

### Calculation of l imits for blank, d etection & quantification

ELISA tests were evaluated for limit of blank (LOB), limit of detection (LOD) and limit of quantification (LOQ) [30,31]:

- Calculation for LOB: (average of blank) + (1.645 × standard deviation of blank);
- Calculation for LOD: (average of 0.1 ng IgA1) + (1.645 × standard deviation of blank); and
- Calculation for LOQ: (average of blank) + (10 × standard deviation of blank) [30,31].

Raw data from the limit calculations (LOB, LOD and LOQ) were converted to Gd-IgA1 units (U) using the standard curve for statistical analysis.

## Statistical analysis

Statistical analysis was performed on LOB, LOD and LOQ data sets using StatPlus for MacIntosh, from AnalystSoft. p-values were calculated using one-way ANOVA.

## Results & discussion

Colorimetric assays are limited by the detection capabilities of the instrument. For most spectrophotometers, the lower limit of detection corresponds to an OD of 0.1. In this assay, using biotin-labeled HPA for colorimetric assessment, the linear range was between 20 and 1.03 ng of standard Gd-IgA1, without compromising the lower limit of spectrophotometric detection range (Figure 1A). Conjugation of HPA to acridinium created a more sensitive readout of Gd-IgA1. Multiple dilutions of HPA–acridinium were tested with serial dilutions of Gd-IgA1 (0.5–10 ng). The results showed good linearity using 1:200, 1:400, 1:600 and 1:800 dilutions of HPA-acridinium (Supplementary Figure 1A, B & C, and Figure 1B). To detect Gd-IgA1 using ≤1 ng of IgA1, further optimization was required. As shown in Tables 1 & 2, 1:600 dilution of HPA–acridinium provided the best sensitivity, with 0.1 ng of Gd-IgA1 falling above the values of blank plus standard deviation. The colorimetric assay was below 0.1 OD at 1 ng of Gd-IgA1 after correction for background, which falls below the normal cutoff for the lower limits of spectrophotometric detection (Tables 1 & 2).

This new chemiluminescence assay can detect standard Gd-IgA1 with a significantly increased sensitivity, by approximately a log-fold, from 1 ng to approximately 0.1 ng Gd-IgA1 for colorimetric vs chemiluminescence assay (Figure 1A & B, Tables 1 & 2). To assess the limit parameters of the ELISA tests, we used a constrained model of methodological analysis for LOB, LOD and LOQ. The LOB values for colorimetric and chemiluminescence (1:600 dilution) assays were 4.07 U vs 1.33 U Gd-IgA1, LOD values 4.27 U vs 1.66 U Gd-IgA1, and LOQ 7.25 U vs 3.16 U Gd-IgA1 (regression analysis based on data from Table 3 and Figure 1A & B). These data show that HPA–acridinium had significantly lower LOB (p < 0.01), LOD (p<0.01) and LOQ (p<0.01) values compared with those for HPA–biotin.

Low concentrations of IgA1 present a significant hurdle in studies with primary cells (i.e.,PBMCs or their subsets; Supplementary Table 1A). A secondary variable is the reactivity of IgA1 with HPA lectin, based on the degree of galactose deficiency. The amount of serum IgA1 that is galactosedeficient is relatively low in healthy individuals, thus producing low spectrophotometric or chemiluminescence values. Therefore, cell cultures of PBMCs from healthy individuals provide suitable specimens to test sensitivity and reproducibility of Gd-IgA1 assays. If successful, this new method would enable investigators to interrogate mechanisms of Gd-IgA1 production in primary cells, a process critical for validating findings from genome-wide association studies and confirming data from studies using immortalized IgA1-producing cells.

Next, we compared the two methods (HPA–acridinium and HPA–biotin) for detection of Gd-IgA1 produced by immortalized IgA1-producing cell lines. Serial dilutions of IgA1 secreted by the cultured cells were assessed. Gd-IgA1 levels were reliably detected in the chemiluminescence assay compared with the colorimetric test, especially at lower concentrations of IgA1 (Tables 4 & 5). In the colorimetric assay, the lower limit of the spectrophoto-metric instrument (OD < 0.1) was especially problematic, with many samples below the detection limit. When LOD cutoff was applied, the colorimetric assay was not adequate for detection, even at the higher concentrations of IgA1. The increase in sensitivity using chemiluminescence assay improved accuracy in assessing amounts of Gd-IgA1 in samples with low concentration of IgA1.

IgA1 production by PBMCs was 1–2 log-fold lower compared with that of immortalized IgA1-secreting cell lines, despite using ~1 log-fold more cells (Supplementary Table 1A & B). To maximize the amount and concentration of IgA1 from cultured PBMCs, the entire population of isolated cells was used and incubated in a smaller volume (0.5 ml vs 1.5 ml for the EBV-immortalized cell lines). The need for high cell density is due, in part, to the low number of IgA1-producing cells in PBMCs. As primary IgA1-producing cells are short-lived, subcloning and expanding these cells is likely not a viable option for increasing IgA1 production.

Media from cultured PBMCs were assessed for Gd-IgA1 using HPA–acridinium and HPA–biotin. As expected, the chemiluminescence assay detected Gd-IgA1 using smaller amounts of total IgA1 compared to colorimetric test (Tables 6 & 7). Based on our LOD limit calculations, data for the colorimetric assay did not meet the cut-off criteria. Typically, for clinical applications, LOQ values would represent the lower limit but, for research purposes, LOD value is an appropriate indicator.

As shown in Supplementary Table 1A & B, IgA1 production by cultured PBMCs was low. Moreover, the variable degree of galactose deficiency in IgA1 secreted by PBMCs from different donors led to low detection signals in some samples (Tables 6 & 7) [10]. The donors of PBMCs in this study were all healthy individuals. Consequently, we would expect that relative degree of galactose deficiency of IgA1 produced by their PBMCs would be lower compared with that of IgA1 from cells from IgAN patients. These samples were thus ideally suited to test the lower limits of the new assay.

This assay used neuraminidase to remove sialic acid from the captured IgA1 to enable detection of total Gd-IgA1. A variant of the assay can compare reactivity with and without neuraminidase, assessing the extent of sialylation of IgA1 from different individuals and cohorts, because HPA binding to galactose-deficient GalNAc is blocked by sialylation (Supplementary Figure 2). This would provide critical information about the functional characteristics of sialylated Gd-IgA1 in pathogenic immune complexes. There is evidence of increased α2,6-sialylation and decreased α2,3-sialylation of circulatory IgA1 from IgAN patients compared with that from healthy controls [18,21,23,29]. The α2,6-sialylation of GalNAc generates a sialyl-Tn antigen, a process catalyzed by ST6GalNAcII, an enzyme overexpressed in IgA1-producing cells from blood of patients with IgAN [21,23]. It has been speculated that premature sialylation of GalNAc by ST6GalNAcII could prevent addition of galactose, thus leading to elevated Gd-IgA1 production [21,23,29,32].

The origin of Gd-IgA1 in the renal immunodeposits in IgAN patients is unclear. Studies show that IgA1 in both the deposits and circulating immune complexes is polymeric, that is, containing J-chain that binds two or more monomers. By contrast, most circulatory IgA1 is in monomeric form, with only ~10% polymeric. It is thought that the monomeric circulatory IgA1 is derived from the bone marrow [33,34]. Polymeric IgA1 is typically produced by plasma cells in mucosal tissues. Notably, IgA1-producing cells derived from PBMCs of patients with IgAN produced elevated amounts of polymeric IgA1 that exhibited a high degree of galactose deficiency, possibly indicating their mucosal origin or homing [21]. This mucosal-tissue connection is consistent with clinical observations: synpharyngitic visible hematuria at disease onset and slower progression of IgAN after tonsil-lectomy in some populations [35–37]. An alternative hypothesis proposed aberrant homing of polymeric Gd-IgA1-producing cells to the bone marrow [38,39]. This assay can detect small amounts of Gd-IgA1, which may help discern the origin of Gd-IgA1-producing cells and their mucosal tissue and/or bone-marrow connections.

The use of chemiluminescence assays in clinical laboratories has increased over the last decade, due to their enhanced sensitivity, linearity and precision compared to that of traditional colorimetric assays [40–43]. Although Gd-IgA1 blood levels can predict disease progression, further validation of this biomarker is necessary before clinical use of this chemiluminescent assay [15,21]. In summary, the greater sensitivity of this new assay will allow researchers to quantify Gd-IgA1 present in fluids in much smaller amounts than is currently possible. This advance will enable mechanistic and translational studies of IgA1 *O*-glycosylation in a robust fashion between populations of IgAN patients and healthy controls.

## Supplementary Material

Supplemental Data

## Figures and Tables

**Table 1.** Raw data for standard Gd-IgA1.

Measurement	Dilution	1 ngGd-lgAI	0.5 ngGd-lgAI	0.1 ngGd-lgAI	Blank
Relative light units	200	1891 ± 403	1590 ± 443	1246 ± 442	1159 ± 388
	400	1899 ± 267	1462 ± 407	1206 ± 214	1171 ± 226
	600	1020 ± 167	828 ± 86	690 ± 76	530 ± 106
	800	783 ± 149	629 ± 98	451 ± 83	451 ± 109
OD 490 nm	100	0.460 ± 0.040	0.414 ± 0.031	0.374 ± 0.083	0.371 ± 0 043

Raw data from dilutions of *Helix pomatia* (HPA)–acridinium (relative light units) and HPA–biotin (OD 490 nm) at low amounts of Gd-IgA1 (0.1–1.0 ng). Dilution refers to fold-dilution of HPA stock solution.

Gd-IgA1: Galactose-deficient IgA1; OD: Optical density.

**Table 2.** Raw data for standard Gd-IgA1 normalized to blank.

Measurement	Dilution	1 ng Gd-lgA1	0.5 ng Gd-lgA1	0.1 ng Gd-lgA1
Relative light units	200	732 ± 560	431 ± 589	88 ± 588
	400	728 ± 350	291 ± 466	35 ± 311
	600	489 ± 198	298 ± 136	160 ± 130
	800	332 ± 185	178 ± 147	−0.4 ± 137
OD 490 nm	100	0.089 ± 0.059	0.043 ± 0.053	0.003 ± 0.093

Raw data corrected for blank signal for low amounts of Gd-IgA1 (1.0, 0.5 and 0.1 ng). N = 16 for *Helix pomatia* (HPA)–acridinium and HPA–biotin. Dilution refers to fold-dilution of HPA stock solution.

Gd-IgA1: Galactose-deficient IgA1; OD: Optical density.

**Table 3.** Detection limits for colorimetric and chemiluminescence Gd-IgA1 assays.

Measurement	Dilution	LOB	LOD	LOQ	Blank
Relative light units	200	1797	1884	5038	1159 ± 388
	400	1543	1895	3805	1171 ± 226
	600	705	864	1590	530 ± 106
	800	631	808	1543	451 ± 109
OD 490 nm	100	0 442	0.465	0.799	0.371 ±0.043

Determination of LOB, LOD, and LOQ for chemiluminescence and colorimetric *Helix pomatia* (HPA) assays. Table shows LOB, LOD and LOQ for different dilutions of HPA–acridinium and HPA–biotin, calculated from blank data and low amount of standard Gd-IgA1 (as detailed in Methods). N = 16. Dilution refers to fold-dilution of HPA stock solution.

Gd-IgA1: Galactose-deficient IgA1; LOB: Limit of blank; LOD: Limit of detection; LOQ: Limit of quantitation; OD: Optical density.

**Table 4.** Colorimetric Gd-IgA1 assay using IgA1 secreted by immortalized IgA1-producing cells.

Cell line	Gd-lgA1 (U)/(OD 490 nm)				
	50 ng IgA	25 ng IgA	12.5 ng lgA	6.25 ng IgA	3.125 ng lgA
1	–/(0.29)	–/(0.10)	–/(0.08)	–/(0.05)	–/(0.04)
2	–/(0.46)	–/(0.18)	–/(0.09)	–/(0.05)	–/(0.06)
3	–/(0.17)	–/(0.06)	–/(0.04)	–/(0.03)	–/(0.01)
4	–/(0.23)	–/(0.11)	–/(0.07)	–/(0.03)	–/(0.02)
5	–/(0.47)	–/(0.22)	–/(0.09)	–/(0.03)	–/(0.05)
6	–/(0.26)	–/(0.12)	–/(0.06)	–/(0.02)	–/(0.02)

Gd-IgA1 detection over a range of IgA1 amounts using the colorimetric assay and optical density values associated with it, after normalization to blank. ‘–’ denotes values OD < 0.1 lower-bound range and/or the limit of detection for the assay.

Gd-IgA1: Galactose-deficient IgA1; OD: Optical density.

**Table 5.** Chemiluminescence Gd-IgA1 assay using IgA1 secreted by immortalized IgA1-producing cells.

Cell line	Gd-lgA1 (U) / (RLU)				
	50 ng IgA	25 ng IgA	12.5 ng IgA	6.25 ng IgA	3.125 ng IgA
1	6.00 (U) / (3496)	2.72 (U) / (1445)	2.73 (U) / (1449)	–/(578)	2.06 (U) / (1031)
2	10.03 (U) / (6016)	4.00 (U) / (2244)	2.81 (U) / (1497)	–/(273)	–/(289)
3	4.48 (U) / (2544)	2.42 (U) / (1254)	–/(453)	–/(335)	–/(227)
4	3.99 (U) / (2239)	1.83 (U) / (886)	2.38 (U) / (1231)	–/(302)	–/(249)
5	7.37 (U) / (4354)	3.13 (U) / (1701)	2.85(U) / (1526)	–/(623)	–/(301)
6	4.18 (U) / (2355)	1.85 (U) / (901)	1.11 (U) / (436)	–/(352)	–/(562)

Gd-IgA1 detection over a range of IgA1 amounts using the chemiluminescence assay. ‘–’ denotes values below limit of detection for this assay.

Gd-IgA1: Galactose-deficient IgA1; RLU: Relative light units.

**Table 6.** Colorimetric Gd-IgA1 assay using IgA1 secreted by cultured peripheral-blood mononuclear cells.

Donor	Gd-lgA1 (U) / (OD 490 nm)		
	12.5 ng IgA	6.25 ng IgA	3.125 ng IgA
A	–/(0.43)	–/(0.19)	–/(0.08)
B	4.90(U) / (0.52)	–/(0.21)	–/(0.09)
C	–/(0.47)	–/(0.19)	–/(0.09)
D	–/(0.41)	–/(0.2)	–/(0.06)
E	–/(0.45)	–/(0.19)	–/(0.05)

Peripheral-blood mononuclear cells cultured *in vitro*. Gd-IgA1 assays with a range of IgA amounts using colorimetric assay. ‘–’ denotes values below limit of detection for this assay. Gd-IgA1: Galactose-deficient IgA1; OD: Optical density.

**Table 7.** Chemiluminescence Gd-IgA1 assay using IgA1 secreted by cultured peripheral-blood mononuclear cells.

	Gd-lgA1 (U) / (RLU)		
Donor	12.5 ng lgA	6.25 ng IgA	3.125 ng IgA
A	4.07 (U) / (3417)	4.36 (U) / (3687)	–/(679)
B	4.29 (U) / (3632)	2.67 (U) / (2123)	1.38 (U) / (929)
C	4.31 (U) / (3642)	2.63 (U) / (2083)	–/(755)
D	5.36 (U) / (4612)	–/(810)	–/(254)
E	3.98 (U) / (3331)	1.55 (U) / (1087)	–/(291)

Peripheral-blood mononuclear cells cultured *in vitro*. Gd-IgA1 assays with a range of IgA amounts using chemiluminescence assay. ‘–’ denotes values below limit of detection for this assay. RLU: Relative light units.
