# Supplementary material for: Assay for galactose-deficient IgA1 enables mechanistic studies with primary cells from IgA nephropathy patients
Source: Biotechniques. Author manuscript; Available in PMC 2018 Sep 24. (PMC6152805; doi:10.2144/btn-2018-0042)

| **Supplementary Table 1 A: IgA1 secreted by immortalized IgA1-producing cell lines from six donors** | | | |
| --- | --- | --- | --- |
| **Cell Line** | **IgA ng/mL** | **Total IgA (ng)** | **Cells/well (x10^6^)** |
| **1** | **2249** | **3374** | **0.6** |
| **2** | **29883** | **44825** | **0.6** |
| **3** | **16953** | **25430** | **0.6** |
| **4** | **14012** | **21018** | **0.6** |
| **5** | **3141** | **4711** | **0.6** |
| **6** | **19613** | **29419** | **0.6** |
| IgA1 production and cell numbers from immortalized IgA1-producing cell lines. Cell lines were seeded in culture medium at the cell density shown and total IgA1 production was assessed. | | | |

| **Supplementary Table 1 B: IgA1 secreted by cultured PBMCs from different donors** | | | |
| --- | --- | --- | --- |
| **Primary** | **IgA (ng/mL)** | **Total IgA (ng)** | **Cells/well (x10^6^)** |
| **A** | **433** | **217** | **8.85** |
| **B** | **345** | **172** | **11.125** |
| **C** | **297** | **149** | **8.465** |
| **D** | **1020** | **510** | **7.35** |
| **E** | **369** | **184** | **5.65** |
| IgA1 production and cell numbers from primary peripheral-blood mononuclear cells (PBMCs). Primary PBMCs from donors were assessed for IgA1 production, and cell densities were based on the total number of cells collected. | | | |

­­­


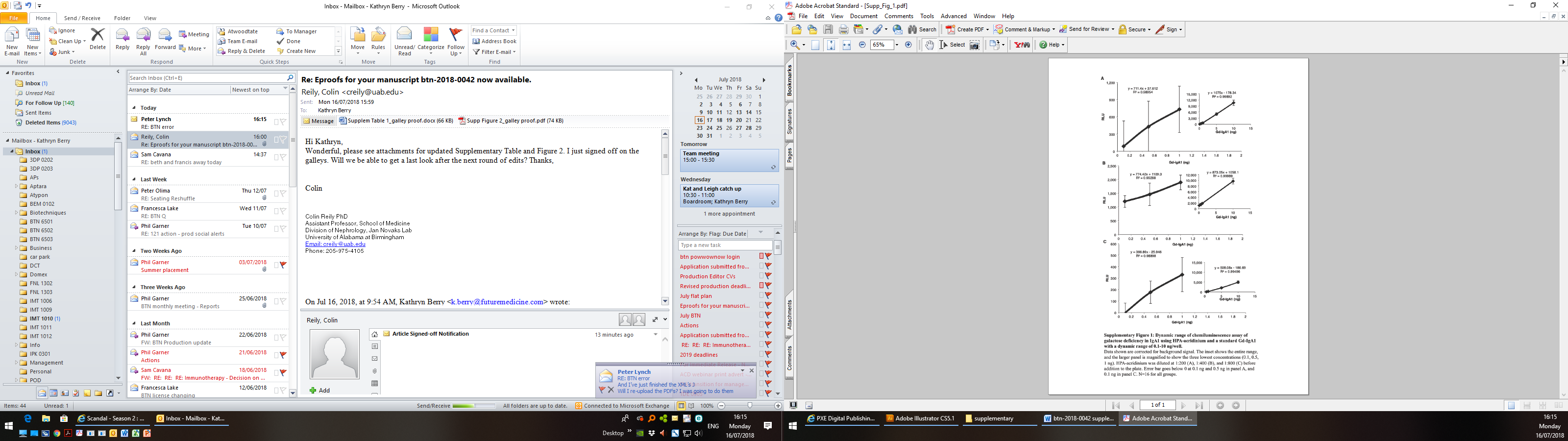


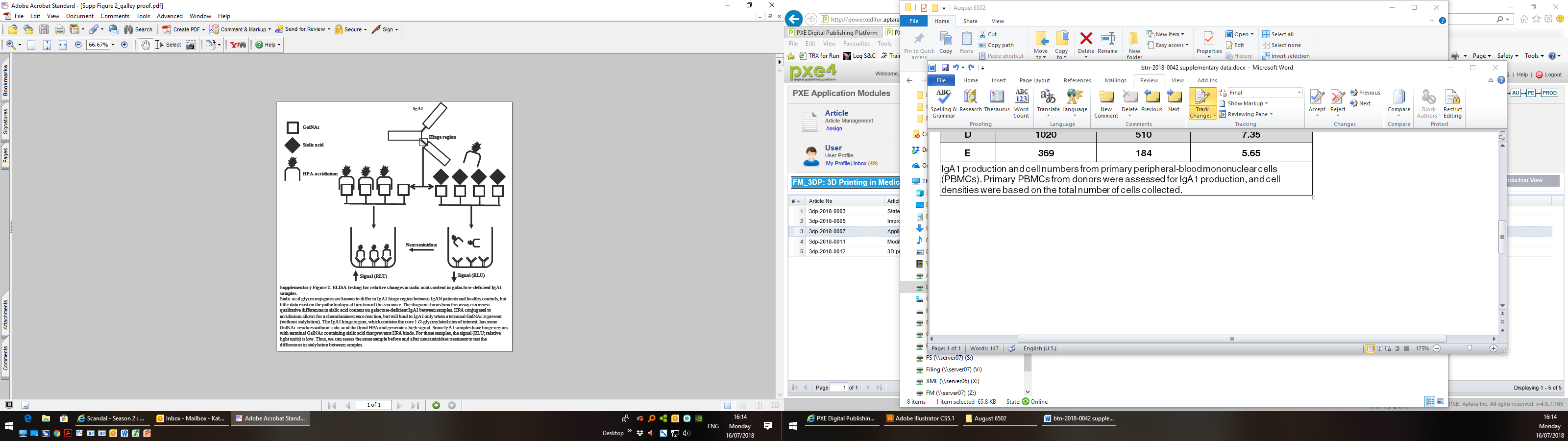

Supplement: Supplemental Data [file NIHMS985424-supplement-Supplemental_Data.docx]
